# National Data on Social Risk Screening Underscore the Need for Implementation Research: The Need for Implementation Research in Social Risk Screening

**DOI:** 10.1001/jamanetworkopen.2019.11513

**Published:** 2019-09-04

**Authors:** Rachel Gold, Laura Gottlieb

**Affiliations:** 1 Kaiser Permanente Center for Health Research–Northwest, Portland, Oregon; 2 OCHIN Inc, Portland, Oregon; 3 Department of Family and Community Medicine, University of California, San Francisco

Social risk screening and documentation in medical settings are recommended by several national medical professional organizations.^1^ These recommendations are based on the increasingly compelling evidence that social risks are associated with poorer treatment adherence, worse health outcomes, and greater costs of care. Systematic screening for patients’ social risks is a key component of providing the data needed to address critical knowledge gaps regarding the role of the health care delivery system in mitigating these detrimental outcomes. But to what extent is social risk screening conducted in clinical settings?

Fraze et al^2^ analyzed data from the 2017–2018 National Survey of Healthcare Organizations and Systems to estimate the prevalence of screening for 5 social risks—food insecurity, housing instability, utility needs, transportation needs, and the occurrence of interpersonal violence—in physician practices and hospitals. The authors also examine organizational characteristics associated with adoption of screening for these risks. According to the responses studied, their results suggest that such screening occurs in a minority of practices and that certain practice characteristics—notably engagement in bundled payment or primary care improvement models—are associated with higher rates of screening adoption.

This is one of the first published studies to describe the uptake of social risk screening across US clinical practices, although it will likely be accompanied by others soon. Multiple national health care surveys have recently incorporated questions about the prevalence of social risk–related activities.^3^ The introduction of these questions is not unexpected given both the recent screening recommendations and new incentives for such screening now offered by some state Medicaid agencies and other payers. Results from this study by Fraze and colleagues,^2^ together with those from other surveys, can help explain whether and how the recommendations and incentives that aim to increase adoption of screening for social risks are associated with on-the-ground clinical practice.

One critical, albeit unsurprising, finding is that reimbursement is indeed associated with uptake of social risk screening. This study by Fraze and colleagues^2^ found that screening for social risks was more common in health care settings that received some reimbursement to support such efforts, either directly or indirectly (eg, through participation in accountable care organizations, receipt of higher levels of Medicaid revenue, state policies that reimburse for interpersonal violence screening, or involvement in bundled payment models). This finding aligns with other research showing that altering incentive structures may enhance the adoption of social risk screening in health care settings.^4–6^ But these findings are just a beginning. Disseminating and sustaining social risk screening will require a deep understanding of how best to structure financial and other incentives to optimally support social risk screening; high-quality research is needed to help design reimbursement models that reliably influence adoption.

It is probable that financial incentives are not the only support that practices will need to implement social risk screening. Recent evidence suggests that practices often encounter multilevel barriers^6,7^ when implementing such screening, some of which may not be addressed through financial support alone. For example, some health care professionals are hesitant to adopt social risk screening if they believe they are unable to address risks reported by patients; others question the need for universal screening.^6^ Ultimately, the adoption of social risk screening is likely to be advanced both by well-designed financial incentives and by addressing potential adoption barriers. A diverse array of such barriers may be encountered; for example, evidence-based interventions may not be available, clinic leadership and staff may lack an understanding that social risk data can help improve care outcomes, and there may be inadequate staff training in how to conduct such screening. The range of adoption rates reported in this study by Fraze et al^2^ likely reflects these and other barriers encountered by respondent practices.

Future research should not only elucidate barriers to the implementation of social risk screening but also surface evidence-based methods for overcoming them. These methods will likely require providing diverse implementation support strategies^8^ tailored to the needs of different health care settings. Implementation science can help us in this pursuit. It is critical to ensure that health care settings seeking to implement social risk screening receive all the implementation support they need for success, or we risk unintended outcomes. For example, although financial incentives for social risk screening might be enough to support adoption of social risk screening in relatively high-functioning clinics, such incentives might be inadequate for clinics that are understaffed or confronting other challenges. In this situation, providing financial incentives alone could lead to greater disparities in the care provided in different settings. Perhaps worse, social risk screening implemented without the use of evidence-based practice transformation strategies might lead some health care professionals or policy makers to conclude that social risk screening is infeasible or ineffective.

This study by Fraze and colleagues^2^ also raises important questions about how to best measure adoption of social risk screening. Large national survey response rates are often low (at 46.5%, this rate was higher than most). Single respondents from 1 institution are unlikely to know about clinical activities across different clinical settings, and there can be wide variation in interpretation of survey questions across respondents. In the present survey, for example, the definition of *routinely* in “Does your practice have a system in place to routinely screen patients for food insecurity,” is unclear. Fraze and colleagues’ findings should be compared with those from the increasing number of national surveys that include similar questions, when results are available. Ultimately, however, all surveys have limitations. Ideally, data from electronic health records and claims data will soon improve our ability to assess the prevalence of social risk–related activities in clinical settings; current efforts by electronic health record vendors to add social risk documentation modules should facilitate progress in this area. However, to date, such standardized documentation of data on social risks and related activities is not yet widespread.

Although momentum for social risk screening is growing nationally, Fraze and colleagues^2^ show that screening across multiple domains is not yet common in clinical settings. Documenting social risk data in health care settings requires identifying ways to implement such screening effectively and sustainably. These findings underscore how much we still have to learn about the types of support needed to implement and sustain these practices.
